# Understanding corporate governance of healthcare quality: a comparative case study of eight Australian public hospitals

**DOI:** 10.1186/s12913-019-4593-0

**Published:** 2019-10-21

**Authors:** Alison Brown

**Affiliations:** Public Service Research Group, School of Business, University of New South Wales, Canberra, Australia

**Keywords:** Healthcare, Governance, Quality, Boards, Processes, Taskwork

## Abstract

**Background:**

Patients are sometimes harmed in the course of receiving hospital care. Existing research has highlighted a positive association between board engagement in healthcare quality activities and healthcare outcomes. However, most research has been undertaken through surveys examining board engagement in a limited number of governance processes. This paper presents evidence of a comprehensive range of processes related to governing healthcare quality undertaken at the corporate governance level. This provides a more detailed picture than previously described of how corporate governance of healthcare quality is enacted by boards and management.

**Methods:**

A comparative case study of eight Australian public hospitals was undertaken. Case studies varying is size and location were selected from two Australian states. Data collection included a review of key governance documentation, semi structured interviews with board members and senior management and an observation of a board quality committee meeting. Thematic analysis was undertaken to identify processes related to key tasks in governing healthcare quality.

**Results:**

Two key tasks in the corporate governance of healthcare quality, evaluating healthcare quality and overseeing quality priorities, were examined. Numerous processes related to these two tasks were found. Case studies, while found to be similar in engagement on previously identified processes, were found to differ in engagement in these additional processes. While generally low levels of engagement in processes of overseeing quality priorities were found, cases differed markedly in their engagement in evaluating healthcare quality processes. Additional processes undertaken at some case studies represent innovative and mature responses to the need for effective corporate governance of healthcare quality. In addition, a group of processes, related to broader governance taskwork, were found to be important in enabling effective corporate governance of healthcare quality.

**Conclusion:**

The work of governing healthcare quality, undertaken at the corporate governance level, is redefined in terms of these more detailed processes. This paper highlights that it is how well these key tasks are undertaken that is important in effective governance. When processes related to key tasks are omitted, the rituals of governance may appear to be satisfied but the responsibility may not be met. Boards and managers need to differentiate between common approaches to governance and practices that enable the fulfilment of governance responsibilities. This study provides practical guidance in outlining processes for effective corporate governance of healthcare quality and highlights areas for further examination.

## Background

Variability in the quality of hospital care is evident through high profile failures and measures of clinical processes and outcomes [1–4]. Reviews of hospital quality failures have indicated a range of factors contributing to preventable patient harm. A common factor identified across reviews is the failure of boards and senior management in overseeing and responding to issues with healthcare quality in their hospitals [5, 6]. Research has increasingly turned toward understanding the contribution of corporate governance to the variability observed in hospital care.

Studies, largely undertaken in the US and UK, demonstrate variable engagement of hospital boards in governance processes such as time spent discussing quality [1, 7–11], placing an item for quality on the board agenda [8, 9, 12] and the regular board monitoring of quality measures [8, 10, 12–14]. The empirical literature further demonstrates evidence of a generally small but significant positive association of healthcare quality measures and board engagement in some governance processes. For example, greater time spent discussing quality at the board [10, 12, 13] and board review of quality performance measures using dashboards or balanced scorecards [12, 13, 15, 16] have both been linked to better healthcare quality.

Research demonstrating an association between governance engagement and healthcare quality measures has highlighted the importance of participation in corporate governance work. However, surveys have used summary descriptions of activities which do not reflect the detailed inner workings of corporate governance. The purpose of this paper is to address this limitation and build on cross sectional survey data and emerging qualitative research to understand in greater detail how corporate governance of healthcare quality is enacted.

This paper describes in detail processes involved in the corporate governance of healthcare quality, herein referred to as healthcare quality governance, from a comparative case study of eight Australian public hospitals. Processes are identified from an analysis of case study data obtained through document review, interview and observation. The argument is made that effective governance is predicated on engagement in a comprehensive range of processes that are integral to effectively implementing important healthcare quality governance tasks. This paper contributes to the literature on healthcare governance in comprehensively detailing these processes. Through presenting a more complete picture of processes, practical guidance is provided to hospitals in reviewing and strengthening the work of governing healthcare quality through fostering greater understanding of and engagement with key processes.

The paper begins by outlining healthcare quality governance tasks and then describes the methods used for an in-depth exploration of two key tasks, evaluating healthcare quality and overseeing quality priorities. Processes related to these two tasks are then identified. Finally, the concept of engagement in healthcare quality governance is re-examined. The paper concludes by urging governance practitioners to differentiate between common approaches to governance, and engagement in a more complete range of processes that further the objectives of healthcare quality governance.

### Healthcare quality governance tasks

Corporate governance is ‘the system by which companies are directed and controlled’ [17]. The dominant model of corporate governance is for organisations to be under the direction of a board [18]. The board model of corporate governance is characterised by board directors acting together, with equal influence, to collectively make decisions about the organisation.

Decisions made by the board are informed and guided by information and advice provided by management. In this paper, the focus is on the corporate governance work of boards and managers in overseeing healthcare quality.

The broader governance literature abounds with descriptions of the boards’ role in setting strategy, assessing organisational performance, and stakeholder engagement [19–22]. Detailed guidance on implementing the board’s role in governing healthcare quality in the peer-reviewed literature is less evident. Board tasks are often described broadly in terms of ‘developing appropriate organisational strategies, incentives and cultures to support the delivery of quality and safety’ [23] and ‘ensur[ing] high quality care’ [24]. Some authors, implicitly or explicitly, reference an agency perspective of governance and discuss the role in terms of quality oversight [12] or accountability [16]. Detailed articulation of healthcare quality taskwork is more commonly found in normative literature and include the following tasks:

- Evaluating and improving healthcare quality performance [1, 16, 25–28]
- Setting and oversight of strategic quality priorities [1, 2, 12, 13, 16, 25–30]
- Promoting leadership and culture [25, 27, 28, 31, 32]
- Ensuring effective systems and processes are in place to maintain and improve quality [1, 28, 31–34].

Similarly, processes related to each healthcare quality task are not described in a comprehensive manner in the literature. Evaluating healthcare quality, is often presented in a simple and passive way. For example, the board ‘reviewed a quality dashboard’ [12] or ‘regularly receives formal reports’ [35]. Reviewing data does not by itself equate to effective evaluation.

A gap in the literature exists in providing a more complete understanding of a range of processes that support the enactment of key tasks. This paper seeks to address this gap through examining how boards and managers undertake the complex work of governing healthcare quality. The study focuses on processes related to two key tasks, evaluating healthcare quality and overseeing quality priorities, reflecting their relative importance in healthcare quality governance and ease of corroborating their related processes through data collection limited to the board and Board quality committee (BQC) and the research methods used. Additional processes, some previously unidentified and some less commonly identified in the literature, have been brought together to portray a more complete picture of how healthcare governance is enacted. The comprehensive exploration of processes, undertaken in this study, provides the basis for re-examining the concept of governance engagement in healthcare quality activities.

## Methods

International empirical healthcare governance research has largely employed quantitative survey methods to develop an initial understanding of engagement in healthcare governance processes and associations with healthcare outcomes. Limitations associated with surveys include the exploration of a small number of briefly described board processes and the use of a single respondent limiting the perspective on governance (see for example [8, 13, 36, 37]). This study uses a comparative case study approach. Case studies allow the use of multiple data sources. The researcher is able to compare and corroborate findings across different data sources to develop ‘a confluence of evidence that breeds credibility, that allows us to feel confident about our observations, interpretations and conclusions’ [38]. The qualitative research methods used in this study are particularly suited to detailed investigation of complex phenomena, such as governance, and provide detailed information to deepen understanding [39]. The approach to case study selection, data collection methods and thematic analysis within the comparative case study design are outlined in this section.

### Sample

Eight Australian public hospitals were recruited as case studies, as part of a broader research project investigating the characteristics of effective governance. Commonwealth health reform in 2011 saw the creation, among a host of other reforms, of local hospital networks (LHNs) comprising single or small groups of functionally connected public hospitals and related services across Australia. However, LHN numbers and governance structures vary between states reflecting a combination of legacy arrangements and state level negotiated reform agreements and legislation. LHNs are governed by boards in most, but not all, jurisdictions. Only LHNs governed by boards with direct responsibility and accountability for governing healthcare quality were relevant to this study. LHNs of this type occurred in sufficient numbers in three states of Australia; Queensland, Victoria and New South Wales. The latter two were selected to be included in the study for practical travel reasons.

Purposive case selection was undertaken to extend the examination of healthcare quality governance to a broader range of public hospitals. Case studies were undertaken in six Victorian and two NSW LHNs. The smaller number of NSW case studies were recruited to enable comparison of state-level contextual factors operating on hospitals. Stratified purposive sampling was employed, with Victorian hospitals stratified according to size and location. This ensured a mix of hospitals of different size and complexity from which to undertake recruitment. Given the similar size of NSW LHNs, a rural and urban site were selected. The overall sample included 4 large multi campus hospitals, 2 medium sized (subregional) rural hospitals and 2 small rural hospitals. All case studies were accredited under national standards. The case or unit of analysis was corporate governance at the hospitals whether it be multisite or single site.

### Data collection and analysis

Data collection was undertaken from July 2016 to April 2017 in the form of document review, interviews and a BQC meeting observation. Evidence confirming the existence of processes already identified in extant literature were first sought. These known processes were later supplemented with additional processes, emerging from the data review, which supported a task. Twelve months’ worth of Board and BQC papers were reviewed at each case study to enable a comprehensive insight into governance activity over a complete annual cycle [40]. Other key governance documents reviewed included terms of reference and planning documents. Systematic documentary analysis was undertaken via a document review template, in the form of a word document, used to summarise the raw data from each case study. The document captured evidence of processes related to key tasks.

Semi-structured interviews were used to clarify and supplement the understanding of governance processes examined in the document review and provided the flexibility to expand on points raised by interviewees [41]. Interviews were requested with the CEO, BQC chair, the senior staff member responsible for healthcare quality and both a board member and clinical executive staff member who attended the BQC. The BQC chair, rather than the Board chair, was interviewed as a detailed exploration of healthcare quality governance at the BQC was required to understand taskwork processes. Thirty-nine participants were interviewed across all case studies, of which 15 were board members, as shown in Table 1. Note the eight case studies are denoted by the nomenclature C1 to C8.

**Table 1:** Profile of interview participants

Position	C1	C2	C3	C4	C5	C6	C7	C8	Total
BQC Chair (board member)	1	1		1	1	1	1		**6**
Board member of BQC	1	1	2	1	1	1		2	**9**
CEO	1	1	1	1		1	1	1	**7**
Director/Manager of quality (DQ/MQ)	1	1	1	2	1	2	1	1	**10**
Director of Nursing (DoN)					1		1		**2**
Director of Medical Services (DMS)	1		1	1				1	**4**
Director of Clinical Program Area (DC)		1							**1**
Total number of people interviewed	**5**	**5**	**5**	**6**	**4**	**5**	**4**	**5**	**39**

Interviews were based on an interview schedule that included questions exploring the work of management and board in governing healthcare quality. Transcribed interviews were imported into NVivo software for analysis. Template analysis, a form of thematic analysis, was employed to code the interview transcripts and involves the development of a codebook to guide the categorisation of segments of text [42]. Interviews were first coded deductively in NVivo according to a codebook developed from a previously developed conceptual framework [43]. Three interviews were initially coded to refine the codebook and then all interviews were coded via the revised codebook. Coding of the data involved two stages, an initial coding followed by a review of coding decisions. This is in line with processes for template analysis described by Brooks et al. [44]. A second inductive and iterative coding process was applied to the initial interview analysis. Material initially coded to framework constructs was then reviewed to identify underlying or emergent themes. Additional coding constructs were created and added to the codebook.

An observation of a single quality committee meeting, ranging in length from 50 to 120 min, was undertaken at each case study. Observations provide a detailed view of meeting practices and dynamics in ‘real time’ that cannot be fully captured by minutes or second-hand descriptions [45]. While the board has overall responsibility for healthcare quality, the BQC, rather than Board, was chosen as the observation site as this is the forum at which most corporate governance work on healthcare quality occurs [46]. Observation notes were analysed for key processes of governance.

Once all data sources had undergone separate initial thematic analysis, a further process was undertaken on the entire data set with a focus on the question of how healthcare quality governance was enacted. Identifying processes involved multiple reviews of the entire data set. All coding and thematic analysis was undertaken by a single researcher, undertaking research for a doctorate.

The study received approval from the Human Research Ethics Committee at the University of Melbourne (Ethics ID: 1646640.2). Informed consent for participation was obtained from all interview participants. Approval was sought from the CEO at each case study for researcher attendance at BQC meetings.

## Results

This study identified a range of healthcare governance processes that enable effective execution of two important healthcare quality governance tasks. In addition, several broader governance processes, undertaken by leaders and influencing how well the Board and BQC addresses their purpose, that have received little attention in the literature, were identified. The processes identified are presented in Table 5 and discussed in this section.

Processes found to be integral to the task of evaluating healthcare quality include; regular robust reporting of a range of data through a range of formats, clear identification of variation and action taken in response and; development and review of a detailed reporting framework, and are described in the following sections.

### Reporting on quality is more than dashboards

All case studies undertook regular healthcare quality reporting, however, the format and content of reporting varied greatly between case studies. Dashboard reporting at the corporate governance level was examined first. Most case studies were found to have dashboards at both the Board and BQC level. There was less variation in the use of indicators informing healthcare quality evaluation observed between case studies when all dashboards were reviewed, than when only the main board dashboard was considered as shown in Table 2.

**Table 2:** Frequency of more common indicators in corporate governance dashboards

Data Category	Indicator Description	Indicator frequency in main board dashboards (*n* = 8)	Indicator frequency in Board and BQC dashboards (*n* = 8)
Safety	Number of serious incidents	3	6
	Medication incidents measures (number or rate)	3	7
	Falls incident measures (number or rate)	4	8
	Pressure injuries measures (number or rate)	5	8
	Patient safety culture	3	3
Effectiveness and Appropriate	*Staphylococcus aureus bacteraemia rate* ^a^	3	4
	Maternity outcomes (including low APGAR, perineal tear, post-partum haemorrhage, caesar rate)	3	4
	Hand Hygiene Compliance	4	6
Acceptable	Patient experience survey (overall experience of care)	4	5
	Timely complaints resolution	4	6
	Met accreditation standards (national or program specific)	3	4
	Met cleaning standards	3	3
Accessible	Access targets	5	6

^a^This indicator was not a state service agreement performance indicator for the four smaller case studies despite being a nationally identified indicator of safety and quality

Between 45 and 94% of dashboard indicators in five case studies were derived from state government service agreements. Service agreement performance indicators were not necessarily seen as being the indicators that were ‘important for us’ (Quality Director, C2).

Greater variation in the types of internally and externally generated reports, collectively to be referred to herein as ‘stand-alone reports’, was found at the corporate governance level. Stand-alone reports informing healthcare quality evaluation routinely scheduled at the Board and BQC as indicated in reporting calendars and agendas are outlined in Table 3.

**Table 3:** Regular healthcare quality reports to Board and BQC

Category	Standalone Report Type	C1	C2	C3	C4	C5	C6	C7	C8
Quality General	Quality Manager or Director report monthly	B	B	QC^a^	B			B	B
Acceptable Care	Consumer stories/case presentation		B/QC		B/QC	B		B/QC	
	Patient experience	B/QC	QC	QC^a^	QC		B/QC		QC
	Compliments and complaints	QC		QC^a^	QC		QC	QC	QC
Safe Care	Incident reports	QC	QC		QC	QC	QC	QC	QC
	Reviews of serious clinical incidents (RCA or clinical reviews)	QC	QC		QC	QC		QC	QC
	Clinical risk profile report	QC	B/QC			B/QC	QC		
	Insurance claims				QC				
Appropriate and Effective Care	Medical Credentialing	QC					B		
	Clinical audit report		QC			QC	QC		QC
	Professional body investigation					QC			
	Other external quality indicator reports^b^	QC	QC	QC^a^	QC	QC	QC		
Culture	Organisational culture	B					B		
Compliance	Accreditation related reports	QC	QC	QC^a^	QC	QC	QC	QC	QC
Annual Operational reports	Clinical risk operational committee annual reports	QC		QC^a^				QC	
	Program/Service area annual reports	QC	QC	QC^a^		QC			
Site Reports^c^			QC	NA	NA		NA	QC	NA
Committee reports	Single operational quality committee minutes	QC		QC^a^		QC			
	Multiple operational quality related committees		QC		QC		QC	QC	QC
	Community Advisory Committee report or similar	QC			QC				

^a^All board members attended the BQC to increase exposure to healthcare quality discussion

^b^(e.g. Health Roundtable, Australian Council on Healthcare Standards, Dental, Aged Care, Dr. Foster)

^c^Site reports from multisite hospitals. NA for this report represents single site hospitals

B = Report presented at Board

QC = Report presented at Board Quality Committee

B/QC = Report presented at both Board and Board Quality Committee

As can be seen from Table 3, the BQC was found to be the main forum for comprehensive exposure to quality reporting. Except for accreditation reports, there was considerable variation in stand-alone report types between case studies. This variation is evident in healthcare quality reports that can be considered fundamental, for example serious incidents and patient feedback. Processes related to developing an appropriate suite of stand-alone reports are important to consider in addition to dashboard reporting. Both reporting approaches are addressed in the next section.

### Reporting for improvement

Healthcare quality data needs to be reported in a way that enables boards to easily identify variation, the actions taken to address unacceptable variation and whether actions are effective in addressing issues. This study found varying engagement in processes that support identifying variation and a quality improvement approach.

Little evidence was found of widespread use of benchmarking, allowing organisational comparisons to be made, regularly presented at the board or BQC. Where benchmarking information was available in external reports, data were often reproduced in board and BQC papers in terms of their relationship to the associated government performance target or their trend in the hospital over time.

Some dashboards used short range trend data (< 12 months) and/or trend arrows to assist in identifying changes in performance since previous reporting periods. Board dashboards, in all five case studies that had a summary indicator table, used traffic light colour coding of results to signal relative performance in relation to a target. Triggering of red or orange flags in relation to unrealistic targets was a concern for many interviewees as explained.

*Your performance is deteriorating in factor X. But when you look at it it's not statistically significant it's just common cause variation and it actually, you know, when you’ve got minimal resource to focus. But what it does is send a red flag to the seniors and the board go ‘oh my god we’re not doing well in this’ but in fact we’re actually, we're OK. (Quality Manager, C5)*

Case studies did not always establish targets for quantitative healthcare measures. When present, the origin and rationale for target setting was not often transparent. The contentious nature of organisational target setting was evident. Views differed as to the value of setting aspirational targets, reflecting a ‘do no harm’ approach that often trigger red flags to which the board can became habituated, versus setting ‘realistic’ targets that acknowledge the inherent risk of acute healthcare delivery.

*Using falls as an example [the quality manager] always explained fairly clearly that yes, there's a certain amount of falls that aren't preventable. So, we can't stop, you know, zero's not really a target unfortunately. So, it's the preventable falls where we really try and focus on and work out. (BQC chair, C4)*

This quote highlights how this hospital responded to the challenge of target setting, with a commonly used indicator, through redefining the indicator and reporting on preventable falls.

In several case studies, dashboard summary healthcare quality indicator tables were supplemented with more detailed indicator information in a complementary report. Detailed indicator reports consisted of graphical or tabular data indicating longer term trends. Along with this, two distinct types of commentary were provided. Firstly, commentary analysing and interpreting the data and identifying unacceptable variation. Secondly, commentary regarding the actions implemented, including, at some case studies, a ‘no action required’ option if data or variation was deemed to be acceptable. This analysis was valuable in translating data into knowledge and supporting board members understanding whilst promoting active reflection and data interpretation by staff.

Variation in the comprehensiveness of data presentation was also seen in stand-alone reports. This variability is highlighted in Table 4 with a review of reports on incidents presented at a corporate governance level, produced by seven case studies. C3 was the exception and reported on select incident indicators (falls and pressure injuries) as part of an indicator dashboard report rather than a more comprehensive standalone report on incidents.

**Table 4:** Format of stand-alone reports on incidents

Format of incident reports	C1	C2	C3	C4	C5	C6	C7	C8
Summary briefing document with background, analysis of data, recommendation/summary of action	✓				✓			
Provision of graphs	✓			✓	✓		✓	✓
Provision of indicator tables	✓	✓			✓		✓	✓
Provision of trended data from 6 to 36 months	✓	✓		✓	✓	✓		✓
Evidence of control limits on some graphs		✓		✓	✓			
Provision of targets				✓		✓		✓
Provision of benchmarked data								
Disaggregated data by site or programs	✓	✓		✓			✓	✓
Narrative analysis of data	✓	✓		✓	✓			
Identification of actions		✓		✓				

Several processes used to identify performance variation, not commonly addressed in the literature, were found in case studies and are seen in incident reporting. Some case studies used graphs to display disaggregated program level data to inform an understanding of variation between programs. Data changes may be due either to common cause variation, non-statistically significant variation that affect all results in a stable process or significant cause variation, due to unusual or unanticipated but potentially identifiable forces [47–49]. A minority of case studies used process control charts to distinguish between significant special cause variation and the normal variation or ‘noise’ in any process. Features such as briefing papers accompanying stand-alone reports were felt to be very useful in highlighting the significance of complex data presented within reports and actions required.

Three case studies presented standalone reports that were thematic reviews of areas, through either clinical risk area annual reports, longer-term analyses of data or reviews of the effectiveness of actions in areas such as incidents or patient feedback. These longer-term thematic reviews shifted the focus from evaluating a narrow reporting period in dashboards to a comprehensive review of trended data seeking to understand contributing factors and intervention effectiveness. As explained,

*At the end of the calendar year let’s analyse all the incident data. Let’s really identify what the issues are and then let’s work out priorities, from a system level. (Quality Manager, C5)*

A few case studies engaged in innovative internal methods of evaluating and detecting variation in healthcare quality. Sophisticated internal or clinical audit mechanisms were employed to identify performance variation and system issues. These mechanisms move beyond familiar compliance and accreditation-related audit processes and represent comprehensive internal reviews of clinical program areas or clinical pathways through the comparison of existing care with internally defined evidence informed standards of care.

Variation in reporting approaches informing an understanding of areas for improvement is strikingly highlighted in the reporting of results from a state department-generated patient experience survey. All case studies within a state receive the same report in an identical format varying only in the identification of organisational details. At C7, no information from the report was presented at the board or BQC level and the hospital was considering how best to use this data to drive improvements. At two case studies the lengthy report was reduced to one or more quantitative indicators that were also government performance agreement indicators. In one of these case studies this was a single indicator subject to a departmental pricing for quality incentive. This contrasts with the remaining five case studies that provided the entire report and in four case studies this was accompanied by a briefing paper summarising the issues and actions taken.

Variation in understanding what constitutes important governance-level information in relation to patient experience is evident and reflects different objectives in presenting data. Comprehensive reporting of state patient experience survey results with briefing papers highlights case studies using reports to inform an understanding of current areas of strength and areas for improvement reflecting a quality improvement focus. In contrast, case studies that reported a few performance agreement indicators have a compliance focus.

### A quality and safety reporting framework

All case studies made incremental changes to reporting through discussions arising at meetings. These iterative discussions were valuable in refining reporting to reflect the current context ‘for the board that we’ve got and for the situation that we’re in’ (Medical Director, C3). Some case studies supplemented incremental data review with formalised processes. Formal review processes were facilitated by detailed annual calendars of reporting and/or detailed indicator frameworks that made transparent targets, their derivation, and the rationale for changes to indicators or targets. These documents were then periodically reviewed and formally endorsed by Board. The documents represent a reporting framework that give a Board a clear helicopter view of data reporting across dimensions of quality, program areas, quality systems, clinical risk areas and sites within a hospital network. This approach contrasts with C8, where no formalised data review was evident,

*We’re just presenting what has historically been presented for the last couple of years. We haven't had a discussion on are there other things the board wants to be seeing and I don't think we've really sat down as an executive and discussed what else should we be providing. (CEO, C8)*

A formal approach to data selection was aided by the process of developing a quality definition. The challenge ‘to work out what actually quality is’ (BQC chair, C6) was experienced not only by board members, at some case studies, but by management.

*It's a challenging area to say the least, about getting that governance right around safety and quality. Cos it’s, it’s sometimes hard to put your finger on what it is. (CEO, C8)*

Definitions of healthcare quality that describe potentially measurable clinical process and outcome categories, such as safe and effective, were found to assist managers and board members to understand the elements that make up quality healthcare and made apparent the broad range of data needed to inform quality evaluation.

Formal and informal mechanisms for board engagement in data selection, endorsement and review are both important in ensuring effective evaluation of healthcare quality. However, formal mechanisms involving the use of reporting frameworks were less commonly used.

### Oversight of healthcare quality priorities

Seven case studies had priorities in their strategic plan for addressing existing healthcare quality couched broadly in terms similar to ‘meeting or exceeding standards of care’ or ‘improving the quality of patient care’. These were seen as being ‘a bit loose’ (CEO, C4). As explained,

*While the directions are probably OK, the detail underneath them about actually being much more explicit in what we’re actually looking to achieve [is missing]. (CEO, C 1).*

The lack of specific strategic healthcare quality priorities seen at most case studies creates a vacuum where staff and board members find it hard to articulate and operationalise strategic initiatives. The need for specific, measurable quality priorities was acknowledged by several interviewees.

*We’ll actually [in the future] put a smart objective together around particular focus areas and that certainly is the stuff that you see in the NHS and where they’ve actually been very targeted around the things that they will focus on. (CEO, C1)*

*If I said we are going to eliminate sepsis, hospital acquired sepsis in the acute hospital by June 2017, people know what it means. Yet, where to me 'providing the right care' … it's a catch all phrase. But there's no specific thing that if you walked around and spoke to everybody and said what are the safety and quality goals for this year. They wouldn't know. (Quality Manager, C5)*

Mechanisms for cascading quality priorities from strategic plans to subordinate governance planning mechanisms included operational plans or a standalone quality plans. Broad strategic quality priorities were cascaded into these plans as a catch-all for a range of government service agreement priorities or other emergent external requirements. Specific quality priorities were seen to be driven externally because external priorities were often ‘more specific’ (Quality Director, C2) than internal strategy.

**Fig. 1:**
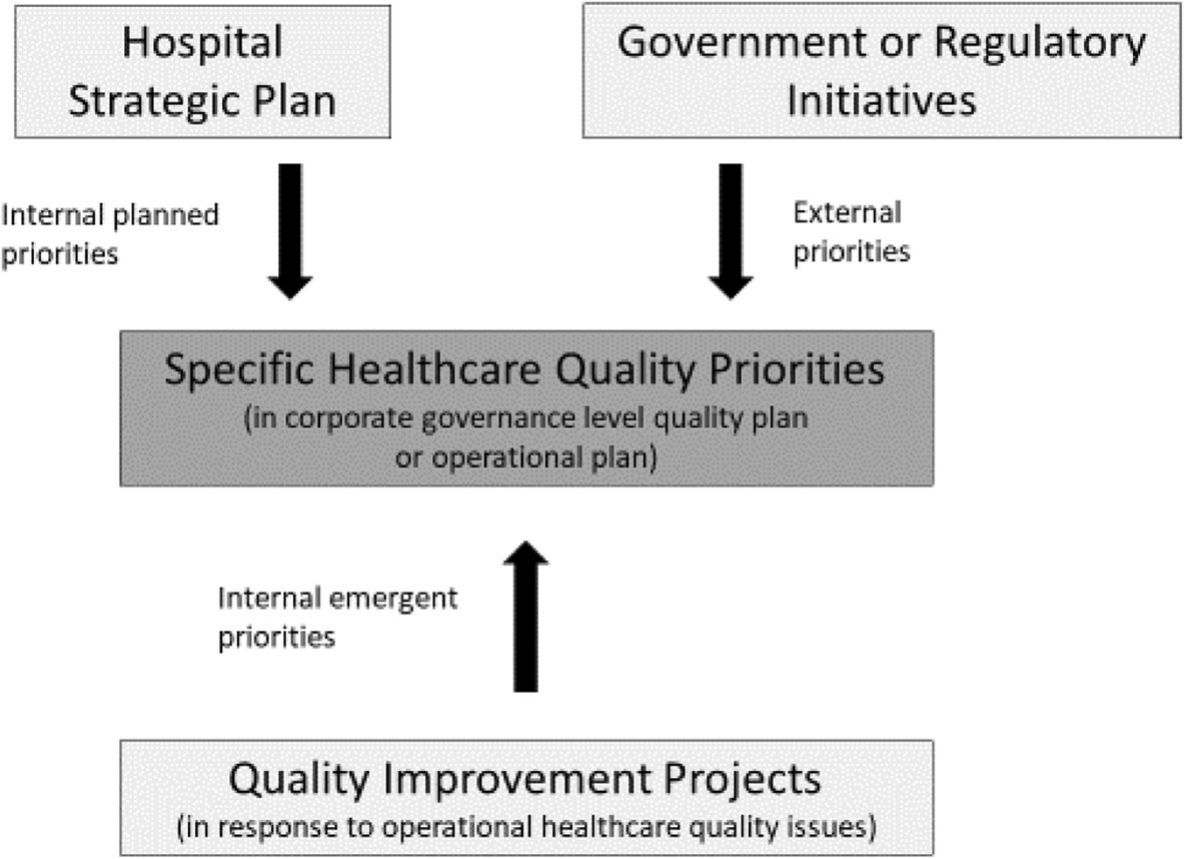
Influences on corporate governance healthcare quality priorities

Broad strategic quality priorities, while useful for maintaining flexibility, were found to be a barrier for priorities being operationalised and reviewed at board level. Only half the case studies reported on progress with strategic priorities at the board and less than half reported on progress with quality priorities at the BQC. Reporting on progress was often through a range of healthcare quality data and KPIs selected to reflect broad strategic directions. Only three case studies had evidence of specific measurable quality priorities identified for which progress could be evaluated. The influences on the development of specific healthcare quality priorities are outlined in Fig. 1.

Most case studies had evidence of substantive quality improvement initiatives being undertaken at an operational level, yet these internal emergent priorities were frequently not made transparent at board level. Only three case studies had corporate governance level planning mechanisms in place that captured and made transparent both planned and emergent priorities. Assessing progress with quality priorities was therefore limited by at least two factors; the lack of specific measurable quality priorities and the lack of transparent reporting on quality priorities at the board or BQC level.

### Governance processes

Additional processes related to broader governance activities that are important in supporting oversight of healthcare quality were identified in this study. These processes, undertaken by leaders, influence how well a Board and BQC understand and enact healthcare quality governance tasks and have received little attention in the literature. Processes include board and committee orientation and skill development, agenda setting, reviewing data reporting and reviewing governance effectiveness through terms of reference and board and committee evaluations (see Table 5).

**Table 5:** Summary of processes related to key healthcare governance tasks

Evaluating Healthcare Quality Processes	Overseeing Quality Priorities Processes	Governance Processes
Processes of selecting healthcare quality data: • Board endorsed definition of healthcare quality exists that identifies measurable categories of quality • Conceptual categories used to structure quality reporting • Board and BQC calendar or schedule that identifies main quality reports and activities • Detailed board and BQC dashboard indicator framework • Periodic scheduled management and board review of reporting content	Strategic quality priority processes: • Strategic priorities addressing quality healthcare • Limited number of specific strategic priorities for improving quality healthcare	Governance processes: • Orientation and skill development • Agenda setting • Reviewing reporting framework • Reviewing governance effectiveness
Reporting processes: • Regular reporting at board and BQC • Dashboard/s indicators reflecting a range of dimensions of quality • Periodic (e.g. annual) thematic standalone quality reports addressing clinical risks, quality systems and program areas • Periodic (e.g. annual) thematic operational quality committee reports	Operationalising quality priorities: • Mechanism for cascading strategic priorities at governance level into subordinate plan • Subordinate governance plan incorporates quality priorities from all sources (planned and emergent)	
Identifying performance variation processes: • Key quality indicators presented with analysis and action implemented, (including no action) • Quantitative data presented graphically with trends, agreed targets or acceptable limits or benchmark comparison • Data disaggregated to reflect program level, where possible • Internal and external reports provided with summary briefing document with background, analysis of data and issues and action • Periodic longer term thematic analysis to identify causes of variation (e.g. incidents, patient feedback or experience) • • Internal methods of performance assessment against evidence-based standards in areas of clinical risk	Monitoring progress processes: • Measurable quality strategies at a governance level • Regular reporting on progress with quality strategies at a governance level	
Action identification processes: • Data analysis and system level action in response to all quantitative and qualitative data • Mechanisms for tracking implementation and effectiveness of action that arise out of data review		

Case studies were seen to vary in their provision of, and comprehensiveness of, board member orientation. Four case studies provided board member orientation that included content specific to healthcare quality and two of these provided a one to one meeting with the quality manager or executive. Structuring meeting agendas and papers is a leadership process that assists effective and efficient running of meetings. At three of the case studies there was evidence of the BQC chair working closely with, or being guided by, the senior quality staffer convening the meeting to shape the agenda and information presented. Committee and reporting review processes are important in ensuring meeting processes and reporting are satisfying the BQCs’ key tasks but occurred at only five case studies.

### Tasks and processes

All processes found to be important in supporting the effective governance of healthcare quality are outlined in Table 5.

## Discussion

Through an in-depth exploration of multiple case studies, a range of processes related to two healthcare quality governance tasks have been identified. The need for regular, robust and timely board reports to inform the evaluation of healthcare quality is a key activity identified in the literature [11, 30]. The extant literature has focused on the presence (and to a lesser extent the content) of board dashboards (see for example 12, 15). This study found the main board dashboard is not a reliable indicator of healthcare quality data reported at the board level for two main reasons. Firstly, the board dashboard is not the only dashboard seen at a corporate governance level and secondly, dashboards reflect a limited range of information, often with a focus on indicators derived from state department of health performance measures. This finding reflects that of Weggelaar-Jansen et al. [50] who found that hospital dashboards focus on easily available external quantitative data. Serious incident and infection measures, which are commonly used departmental performance measures, often represent relatively infrequent events and are generally less sensitive indicators of changes in healthcare quality, unless occurring frequently at a particular hospital [4].

Goeschel et al. [51] highlight, a limited focus on the presence or content of dashboards does not inform what other information the board gets or how this information is used. Greater variation was found in the types and presentation of internally and externally generated reports. Processes around both the development of both dashboards and stand-alone reporting are therefore important to explore when examining the task of healthcare quality evaluation.

Mechanisms for identifying variation in healthcare quality such as trending and benchmarking have been examined in previous surveys [12, 15, 16] but relatively little attention has been paid to how well these mechanisms are used. In this study, the traffic light coding, short-term trend data and trend arrows, that were used frequently in dashboards did not assist in identifying the nature of variation occurring. When discrete time point data is presented it is more than likely that figures in the previous reporting period will be higher or lower [52]. Changes in discrete time point results can trigger red or orange flags in response to non-significant or common cause variation or the normal ‘noise’ within a process [53, 54]. Red and orange flags triggered in relation to unrealistic targets can draw the board into unnecessary discussion of common cause variation [47, 52]. The study also found the use of aspirational no harm targets for some indicators were a problem in frequent triggering of dashboard red flags to which boards members can become habituated. Short term incremental targets are more appropriate for regular progress monitoring, with zero harm targets reserved for aspirational goals [26].

The use of a detailed indicator report complementing a summary indicator table was found at case studies with a more mature and comprehensive approach to dashboard reporting. The value of this approach has been noted in recent literature [50]. Longer term trends portrayed in graphs in these more detailed reports allow ready identification of data patterns and are better at identifying variation for some indicators [54]. Similarly, the use of data disaggregated at clinical area level is useful as complication rates vary by speciality and aggregated data can hide underperformance in specific program areas [4]. The use of clinical area, or even clinician level comparative data, may however be limited in smaller hospitals by smaller sample sizes. The presentation of longer-term trends, the use of process control charts, disaggregated data and commentary analysing data and identifying actions are useful formats in highlighting unacceptable variation in dashboards.

The study demonstrated greater variation in the types and content of stand-alone reports, than dashboards. The findings from both incident and externally generated patient experience reports show diverse approaches to data use, identification of variation and actions. Case studies varied in using data to provide assurance only on compliance indicators to more sophisticated methods of detecting and analysing healthcare quality variation to support a quality improvement approach. This finding is echoed in the research of Jones et al. [46] who found higher performing boards use data for quality improvement, rather than assurance.

This study highlights the importance of carefully selecting data and reports to inform an evaluation of healthcare quality. While selecting and endorsing data used to inform healthcare quality evaluation is a key board process identified in the literature [7, 11, 55, 56] the literature has largely been silent on how this happens. This study found developing a data reporting framework was an important process in identifying the types of data needed, either in standalone reports or in indicator dashboards. A reporting framework can, through referencing quality dimensions, make apparent the need to identify a broad range of data to inform the task of evaluating healthcare quality [57, 58]. Additional healthcare quality governance tasks, such as oversight of quality priorities, can also be reflected in a reporting framework as shown in Table 6. Board and committee calendars can then be generated from the framework, with the addition of any other specific governance tasks as described in charters or terms of reference.

**Table 6:** Example of approach to developing reporting framework

Tasks	Type of reporting required	Where is this report presented, format and frequency		
		Board	Board Quality Committee	Operational Quality committee
Healthcare quality tasks				
Evaluating healthcare quality through reviewing if care is:				
• Safe				
• Person-centred				
• Effective				
• …				
Overseeing quality priorities				
Promoting leadership and culture				
Ensuring effective quality systems				
Governance tasks				
Ensuring effective board/committee				

The disparity in approach to evaluating healthcare quality at cases, reflects the extent to which a thoughtful and mature governance approach to data selection, data analysis and monitoring action was undertaken. Case studies seeking information to provide insight into system level issues and action to inform quality improvement, engaged in a number of additional processes not previously discussed in the literature.

Literature descriptions of the task of overseeing healthcare quality priorities have a focus on boards establishing strategic goals for quality [1, 3, 7–9, 12, 59]. Processes of cascading priorities throughout the organisation [15, 60] and monitoring strategic progress [1, 61] receive less attention. While additional processes were found that supported this task in this study (see Table 5), there was generally low engagement in all quality priority oversight processes.

Planning processes commonly incorporate elements of both planned, deliberate priorities and emergent priorities formed in response to newly identified risks or opportunities [62, 63]. The case studies used broad strategic priorities to accommodate the abundance of quality priorities arising from various regulatory bodies. The multitude of external priorities creates ‘priority thickets’ [2] from which services struggle to find space for internally planned strategic quality priorities. The articulation of broad strategies can therefore be seen as an astute management strategy to keep options open to accommodate emergent external priorities [62]. However, the need for more specific strategic healthcare quality priorities, to set a clear direction throughout the hospital, was apparent from interviewees.

Most case studies demonstrated an understanding of the need for healthcare quality priorities as evidenced by their presence in strategic plans. However, moving beyond symbolic acknowledgement and cascading specific planned priorities or elevating internal emergent priorities into quality planning mechanisms overseen by the governing body was not apparent in most case studies. This finding reflects the research of Demb [64] who found that boards are less involved in strategy oversight when organisations are more focussed on emergent external priorities, than planned internal strategies. The need for specific quality priorities visible at the board level is also consistent with that of Jones et al. [46] who found that oversight of both planned and emergent strategies is a feature of higher performing boards.

In addition to processes directly related to the two healthcare governance tasks, this study identified a set of governance processes that supported effective leadership and governance. This finding is similar to that of Cornforth [65] who, in a survey of UK charity boards, found that having the right skills, a clear understanding of roles and responsibilities, and board and management that periodically review how they work together, were key factors in explaining variance in board effectiveness.

### Redefining engagement

This paper has identified a range of processes that support the enactment of key tasks in governing healthcare quality. Healthcare quality governance work can now be redefined in terms of these processes (see Table 5). This builds on previous research which identified a limited number of processes. When case studies are compared based on their participation in commonly cited processes, their level of engagement is broadly similar as show in Table 7.

**Table 7:** Comparison of engagement levels based on existing literature

Commonly cited processes	C1	C2	C3	C4	C5	C6	C7	C8
Evaluating Healthcare Quality								
Board quality committee exists [10–12, 15, 16]	✓	✓	✓^a^	✓	✓	✓	✓	✓
Board regularly review quality healthcare performance [8, 10–13, 30]	✓	✓	✓^a^	✓	✓	✓	✓	✓
Board uses a quality scorecard or dashboard [12, 15, 16, 55, 61]	✓	✓	✓^a^	✓		✓	✓	✓
Trending and benchmarking performance [12, 15, 16]	✓	✓	✓^a^	✓	✓	✓	✓	✓
Board Agenda has an item on quality (includes quality agenda item or quality directorate report but excludes BQC minutes) [1, 8, 12, 61]	✓	✓	✓^a^	✓^a^			✓	
Overseeing Quality Priorities								
Board has established or endorsed goals relating to patient outcomes [1, 8, 12, 30, 61]	✓	✓	✓		✓	✓	✓	✓

^a^Through dedicated committee which whole board attends (C3 BQC and C4 Performance Committee)

This is consistent with the findings of Freeman et al. [66] who found that commonly cited governance processes are not useful for discriminating between hospitals. The frequency with which most case studies undertake these processes reflects a form of institutional isomorphism. Mimetic institutional isomorphism is characterised by organisations dealing with uncertainty by adopting pre-existing processes used by peers perceived to be high performing [67]. In much the same way that healthcare boards historically adopted models of governance from the commercial world with the accompanying focus on financial accountability, there has been a similar isomorphism in the area of healthcare quality governance. This has led to a focus on a narrow range of governance processes that have been highlighted in literature and guidelines. These common processes have lost their discriminatory power in evaluating engagement in healthcare quality activities. They do not adequately represent the range of processes that boards need to engage with to effectively enact tasks related to healthcare quality governance responsibilities. The adoption of additional processes, demonstrated at some case studies, represent more mature, and at times innovative, governance approaches. The devolved corporate governance model of hospital governance relies on the assumption that boards and management understand the work of governance. This study indicates that this is not always the case and that taskwork processes need to be clearly articulated in legislation, regulation and guidelines.

Examining the work of boards and senior managers, via qualitative methods in this study, has made visible additional important processes in relation to two key tasks of healthcare quality governance, evaluating healthcare quality and overseeing quality priorities as well as broader governance processes that support a more mature response to executing key governance responsibilities. This finding makes clear that it is not just engagement with taskwork that is important, but the quality of that engagement. Effective engagement is predicated on how well the various processes, comprising a task, are undertaken.

## Conclusion

This paper presents a comprehensive examination of the work of governing healthcare quality in key areas. Data from documents, interviews and observations has been reviewed from eight public hospitals in Australia to identify a range of processes related to two key tasks. The focus on dashboard reporting processes alone in much of the existing literature is not warranted as dashboard data is a small and less variable component of healthcare quality data reported at the corporate governance level. Summary indicator tables found in dashboards were supplemented, in some case studies, with more useful detailed indicator reports and stand-alone reports using a range of formats to identify variation and action. Processes of overseeing quality priorities were underutilised by all case studies and reflect the dominant influence of external quality priorities in setting the agenda in hospitals.

While comprehensive data was collected related to two tasks of healthcare quality governance there are at least two other tasks that require similar exploration including promoting leadership and culture, and ensuring effective quality systems. Further in-depth examination of these tasks would provide evidence of additional processes of healthcare quality governance. This study demonstrates that previous research into taskwork processes, undertaken internationally, while relevant to the Australian context, does not go far enough in describing detailed processes related to taskwork. While valuable data was obtained, in this study, on additional processes it would be useful to undertake further research in different countries to confirm and expand on the current findings.

This study highlights that engagement in taskwork is variable and this can impact on how well healthcare quality governance is enacted. Reporting on a few quality indicators related to performance agreements can provide assurance on compliance requirements. This differs from engagement in a multitude of processes that ensure a range of appropriate data is selected and reported in a format that is easily understood and informs the evaluation of healthcare quality at the corporate governance level. This finding is reflected in recommendations arising from the inquiry into failings at the Mid-Staffordshire Hospital which include the need for careful selection of quality data and the establishment of norms so that poor performance can be identified [6]. Similarly, the presence of quality priorities in a strategic plan does not necessarily ensure their translation into measurable strategies that are cascaded through the organisation and monitored for progress.

When key processes are omitted, the rituals of governance may appear to be satisfied but the responsibility to effectively govern healthcare quality may not be met. Boards and managers need to differentiate between common approaches to governance and effective engagement in a range of taskwork processes that enable the fulfilment of governance responsibilities. The findings from this study provide practical guidance to governing bodies in the execution of two key tasks of healthcare quality governance. Enactment of these healthcare quality tasks is aided by engagement with a set of broader governance processes to ensure the effective working of boards and committees.

## Data Availability

The data analysed during the current study are not publicly available due to ethical confidentiality requirements. Requests for summary de-identified data can be made to the corresponding author.

## Abbreviations

BQC: Board Quality Committee
C: Case Study
LHN: Local Hospital Network
UK: United Kingdom
US: United States
